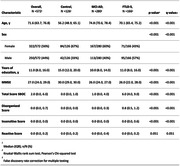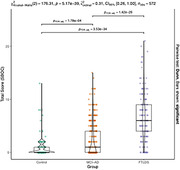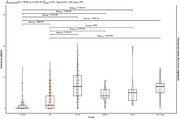# Diagnostic Utility of the Social Behavior Observer Checklist in Neurodegenerative Disease Assessment: insights from a biomarker‐enriched cohort in Spain

**DOI:** 10.1002/alz.092573

**Published:** 2025-01-03

**Authors:** Judit Selma‐Gonzalez, María Belén Sánchez‐Saudinós, Isabel Sala, Sara Rubio‐Guerra, Jesús Garcia Castro, Nuole Zhu, Javier Arranz, Íñigo Rodríguez‐Baz, José Enrique Arriola‐Infante, Lucía Maure‐Blesa, Juan Fortea, Daniel Alcolea, Miguel A Santos‐Santos, Maria Carmona‐Iragui, Alberto Lleo, Ignacio Illán‐Gala

**Affiliations:** ^1^ Sant Pau Memory Unit, Hospital de la Santa Creu i Sant Pau ‐ Biomedical Research Institute Sant Pau ‐ Universitat Autònoma de Barcelona, Barcelona Spain; ^2^ Hospital de la Santa Creu i Sant Pau ‐ Biomedical Research Institute Sant Pau ‐ Autonomous University of Barcelona, Barcelona, Catalonia Spain; ^3^ Sant Pau Memory Unit, Department of Neurology, Hospital de la Santa Creu i Sant Pau, Biomedical Research Institute Sant Pau ‐ Universitat Autònoma de Barcelona, Barcelona, Spain Spain; ^4^ Centre of Biomedical Investigation Network for Neurodegenerative Diseases (CIBERNED), Madrid Spain; ^5^ Sant Pau Memory Unit, Hospital de la Santa Creu i Sant Pau, Biomedical Research Institute Sant Pau, Universitat Autònoma de Barcelona, Barcelona Spain; ^6^ Sant Pau Memory Unit, Hospital de la Santa Creu i Sant Pau, Biomedical Research Institute Sant Pau, Universitat Autònoma de Barcelona, Barcelona, Barcelona Spain; ^7^ Barcelona Down Medical Center, Fundació Catalana Síndrome de Down, Barcelona Spain; ^8^ Hospital de la Santa Creu i Sant Pau ‐ Biomedical Research Institute Sant Pau ‐ Autonomous University of Barcelona, Barcelona Spain; ^9^ Sant Pau Memory Unit, Department of Neurology, Hospital de la Santa Creu i Sant Pau, Biomedical Research Institute Sant Pau ‐ Universitat Autònoma de Barcelona, Barcelona Spain

## Abstract

**Background:**

Patients with neurodegenerative diseases can display early behavioral changes during the clinical assessment that can be captured with the Social Behavior Observer Checklist (SBOC). Nevertheless, the incremental diagnostic utility of this structured observational instrument, when used in conjunction with conventional neuropsychological assessments, has not been ascertained across diverse cultural contexts. We aimed to determine the diagnostic value of the SBOC in large biomarker supported cohort of patients with different neurodegenerative diseases.

**Method:**

Participants from the SPIN cohort underwent extensive diagnostic protocols, including neuropsychological evaluation and cerebrospinal fluid biomarker analysis. Diagnostic categories were assigned based on consensus criteria. We derived cognitive composites (memory, language, executive function, and visuospatial function) by averaging scaled scores measuring parallel neuropsychological constructs. A total SBOC score and three subscale scores (disorganized, reactive, and insensitive subscales) were calculated. We explored the correlation between SBOC scores and cognitive domains. We studied the diagnostic accuracy of SBOC measures by calculating the Area Under the Receiver Operating Curve (AUROC). We compared the AUROC of SBOC‐derived scores and cognitive composites with the DeLong’s test.

**Result:**

The cohort included 572 participants: 126 cognitively healthy controls (HC), 166 with Frontotemporal Lobar Degeneration Syndromes (FTLD‐S), and 280 with Alzheimer’s disease (AD). FTLD‐S encompassed 95 with behavioral variant frontotemporal dementia (bvFTD), 26 non‐fluent/agrammatic, 8 semantic variant primary progressive aphasias, and 37 progressive supranuclear palsy‐corticobasal degeneration spectrum disorders. SBOC scores exhibited modest correlations with executive functions. FTLD‐S patients had higher SBOC scores than HC and AD, with bvFTD patients showing the greatest elevations. The diagnostic accuracy of the SBOC was high for distinguishing FTLD‐S from HC (AUROC = 0.91 for total and 0.94 for disorganized scores) and bvFTD from HC (AUROC = 0.90 for total and 0.92 for disorganized scores), matching the accuracy of cognitive composites. However, SBOC scores did not differentiate between FTLD‐S and AD (AUROC = 0.61) or between FTLD‐S subtypes.

**Conclusion:**

The SBOC is a useful scale that can complement the information obtained from formal neuropsychological testing and help identifying prototypical behaviors associated with different neurodegenerative diseases.